# Dental arch dimensional changes after adenoidectomy or tonsillectomy in children with airway obstruction: A meta-analysis and systematic review under PRISMA guidelines

**DOI:** 10.1097/MD.0000000000004976

**Published:** 2016-09-30

**Authors:** Yanfei Zhu, Jiaying Li, Yanmei Tang, Xiaoling Wang, Xiaochen Xue, Huijun Sun, Ping Nie, Xinhua Qu, Min Zhu

**Affiliations:** a College of Stomatology, Shanghai Jiao Tong University School of Medicine, Shanghai; b Jining Medical University, Jining, Shandong; c Department of Oral and Cranio-Maxillofacial Science; d Department of Orthopaedics, Shanghai Ninth People's Hospital; e Shanghai Key Laboratory of Orthopaedic Implant; f Shanghai Key Laboratory of Stomatology, Shanghai, China.

**Keywords:** adenoidectomy, airway obstruction, children, dental arch, tonsillectomy

## Abstract

**Background:**

Children with severe airway obstruction tend to have a vertical direction of growth, class II malocclusion, and narrow arches. Adenoidectomy and tonsillectomy were recommended for the promotion of balanced dentition growth in these children.

The aim of this study was to determine the effect of adenoidectomy and tonsillectomy on the growth of dental morphology in children with airway obstruction.

**Methods:**

A comprehensive search of the Medline, Embase, Web of science, and OVID databases for studies published through to January 17, 2016 was conducted. Prospective, comparative, clinical studies assessing the efficacy of adenoidectomy, or tonsillectomy in children with airway obstruction were included. The weighted mean difference (WMD) and 95% confidence interval (CI) were used for continuous variables. Forest plots were drawn to demonstrate effects in the meta-analyses.

**Results:**

Eight papers were included in our study. We found that adenoidectomy and tonsillectomy led to a significant change in nasal-breathing in children with airway obstruction. Children with airway obstruction had a significantly narrower posterior maxillary dental arch than children without airway obstruction (WMD = −0.94, 95% CI [−1.13, −0.76]; *P* < 0.001). After surgery, these children still had a significantly narrower dental arch than the nasal-breathing children (WMD = −0.60, 95% CI [−0.79, −0.42]; *P* < 0.001). In terms of dental arch width, malocclusion, palatal height, overjet, overbite, dental arch perimeter, and arch length, a tendency toward normalization was evident following adenoidectomy or tonsillectomy, with no significant differences evident between the surgical group and the normal group. The small number of studies and lack of randomized controlled trials were the main limitations of this meta-analysis.

**Conclusions:**

Following adenoidectomy and tonsillectomy, the malocclusion and narrow arch width of children with airway obstruction could not be completely reversed. Therefore, other treatments such as functional training or orthodontic maxillary widening should be considered after removing the obstruction in the airway.

## Abstract 2

Supplemental Digital Content is available in the text

## 1 Introduction

During child's growth, active immunologic processes may cause a physiological and fluctuating tonsil and adenoid hypertrophy, particularly during the 1st 4 to 6 years of age.^[1]^ Tonsillar and adenoid enlargement are considered the most common contributors of upper airway obstruction in young children.^[2,3]^

The obstruction reduces the depth of oropharynx, thereby leading to lowered posturing of the hyoid bone, which forces the tongue anteriorly and causes a compensatory change in the child's mode of breathing.^[4–7]^ Additionally, the upper airway obstruction and altered breathing pattern may potentially affect the child's dentofacial growth.^[5,8]^ Recently, many institutions have observed altered dental arch development in young children following upper airway obstruction. These observations included increased palatal depth, narrowing of the upper dental arch, increased overjet, and increased anterior open bite and posterior crossbite.^[9–12]^

Appropriate removal of the obstructive factors is considered to be pivotal in the normalization of breathing patterns, the positive promotion of balanced dentition growth, and the enhancement of orthodontic treatment stability.^[13]^ Thus, the idea that adenoidectomy or tonsillectomy could potentially interrupt the development of dentofacial deformity, and partly reverse malocclusion at an early age has been proposed by some cohort studies.^[1,11,14]^ Conversely, some otolaryngologists, or orthodontists, found that the effects of adenoidectomy or tonsillectomy on dental arch growth was limited, and would likely relapse.^[15,16]^ The effect of adenoidectomy and tonsillectomy on dental arch morphology is difficult to predict.

Therefore, the aim of this systematic review and meta-analysis was to summarize the literature regarding adenoidectomy and tonsillectomy treatment outcomes and to verify whether adenoidectomy and tonsillectomy contribute to the normalization of dental arch development in children with airway obstruction.

## 2 Methods

### 2.1 Focused question

We hope to verify the following hypothesis: adenoidectomy or tonsillectomy contributes to the complete normalization of dental arch development in children with airway obstruction.

The population, intervention, comparison, outcome, and study design (PICOs) definition was developed based upon the focused question as follows:

- Population: children with no previous or ongoing orthodontic treatment.
- Intervention: adenoidectomy and/or tonsillectomy.
- Comparison: children with or without upper airway obstruction and not undergoing adenoidectomy or tonsillectomy.
- Outcomes: the primary outcome was: dental arch width; the secondary outcomes were change in breathing pattern, malocclusion, palatal depth, overjet, overbite, dental arch length, dental arch perimeter, and transverse relationship between jaws.
- Study design: prospective clinical comparative study.

### 2.2 Search strategies

An electronic search limited to English was conducted using the Medline, Web of Science, Embase, and OVID databases. All studies published through to January 17, 2016 were included. The reference lists of included studies and relevant reviews were also searched for other potential studies. The detailed search strategies were as follows:

#1 MeSH descriptor: [Child] explode all trees
#2 child^∗^ or paediat^∗^ or pediat^∗^ or adolesc^∗^ or toddler^∗^ or preschool^∗^ or pre school^∗^ or pre-school^∗^ or prepube^∗^
#3 #1 or #2
#4 MeSH descriptor: [Tonsillectomy] explode all trees
#5 MeSH descriptor: [Palatine Tonsil] explode all trees and with qualifier(s): [Surgery - SU]
#6 MeSH descriptor: [Adenoidectomy] explode all trees
#7 MeSH descriptor: [Adenoids] explode all trees and with qualifier(s): [Surgery - SU]
#8 tonsillectom^∗^ or tonsillotom^∗^ or adenoidectom^∗^ or adenotonsillectom^∗^ or adeno-tonsillectom^∗^
#9 MeSH descriptor: [Palatine Tonsil] explode all trees
#10 MeSH descriptor: [Adenoids] explode all trees
#11 tonsil^∗^ or adenoid^∗^ or adenotonsil^∗^
#12 #9 or #10 or #11
#13 MeSH descriptor: [Surgical Procedures, Operative] explode all trees
#14 surger^∗^ or extract^∗^ or dissect^∗^or excis^∗^ or resect^∗^ or operation or remov^∗^ or coblat^∗^ or ablat^∗^ or laser
#15 #13 or #14
#16 #12 and #15
#17 #4 or #5 or #6 or #7 or #8 or #16
#18 MeSH descriptor: [Dental Arch] explode all trees
#19 MeSH descriptor: [Craniofacial Abnormalities] explode all trees
#20 MeSH descriptor: [Dentofacial Deformities] explode all trees
#21 craniofacial^∗^ or cranio-facial^∗^ or dentofacial^∗^ or dento-facial^∗^ or dentoalveolar^∗^ or dento-alveolar^∗^
#22 dental or dentition or upper arch^∗^ or lower arch^∗^ or upper jaw^∗^ or lower jaw^∗^ or maxilla^∗^ or mandib^∗^ or molar width or intermolar width or occlusion or orthodonti^∗^ or malocclusion
#23 #18 or #19 or #20 or #21 or #22
#24 #3 and #17 and #23

### 2.3 Eligibility criteria

The inclusion criteria were as follows: children, studies conducting adenoidectomy or tonsillectomy as a treatment for children with airway obstruction, studies providing data regarding the dental cast measurements, studies with follow-up periods of at least 1 year after surgery, prospective clinical comparative studies, and studies published in English.

Furthermore, the following exclusion criteria were used: animal studies, in vitro studies, case reports, reviews, and studies including participants with craniofacial syndromes or receiving orthodontic treatments before evaluating.

### 2.4 Study selection

Two investigators (YZ and JL) separately reviewed the titles and abstracts for the selection of relevant studies. Studies that could not be excluded definitively upon the basis of the information gleamed from titles and abstracts were analyzed full-text in order to determine inclusion criteria eligibility. If a unanimous agreement could not be reached according to the selection criteria, a discussion was held with a 3rd investigator until an agreement was reached (MZ).

### 2.5 Data extraction

Two investigators (YZ and JL) separately extracted data using a specially designed extraction form. Any discrepancy between the data extracted by the 2 investigators was discussed with a 3rd investigator (MZ). The interreviewer reliability of data extraction was evaluated by the percentage of agreement and value of Kappa analyses. The most complete data with the longest follow-up period were extracted. The following information was extracted from each included study: first author's name, year of publication, country, study design, follow-up period, number and mean age of patients, description of test and control groups, intervention, malocclusion, breathing pattern, dental arch width, other parameters (palatal height, overjet, overbite, transverse relationship between jaws, dental arch length, and perimeter), and the conclusion.

### 2.6 Quality assessment

The Newcastle–Ottawa Scale was used to assess the quality of nonrandomized studies. This scale classified ratings based on three categories: selection, comparability, and (3) outcome in cohort studies. The methodological quality of included studies was evaluated by the number of stars given: total score ≤3, low quality; 4 or 5, moderate quality; and ≥6, high quality; maximum total score was 9.

### 2.7 Data analysis

A meta-analysis would be conducted when 2 or more of the included studies used similar dental cast measurements. The weighted mean difference (WMD) and 95% confidence interval (CI) were used for continuous variables (dental arch width). Forest plots were drawn to demonstrate the effects in the meta-analyses.

Q-tests and *I*^2^ statistics were applied to tested heterogeneity among the included studies (*I*^2^ ≤ 25%: low heterogeneity; 25% < *I*^2^ < 50%: moderate heterogeneity; and *I*^2^ ≥ 75%: high heterogeneity).^[17]^ A fixed-effects model was used as a common measure for a study-specific estimate, while a random-effects model was considered when significant heterogeneity was demonstrated among studies.^[18]^ Visualization of funnel plots was drawn to assess publication biases.

Review Manager 5.3 (The Cochrane Collaboration, Copenhagen, Denmark) was used to conduct the statistical analyses.

As this study is a meta-analysis and systematic review, ethical approval or patient consent was not necessary.

## 3 Results

### 3.1 Literature search

**Figure 1:**
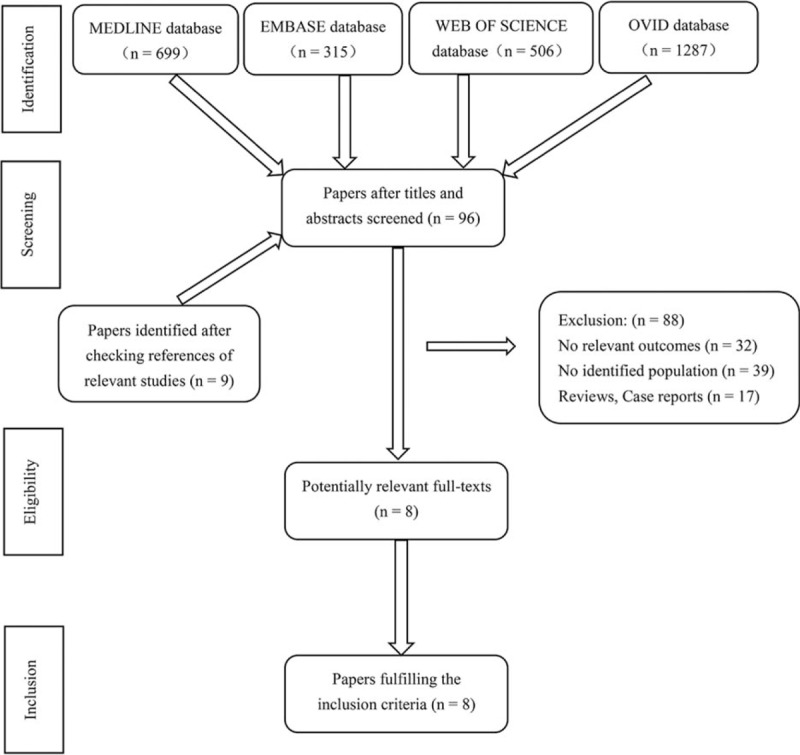
Flow-chart depicting the literature search procedure.

The flow chart of literature search process is presented in Fig. 1. The search yielded a total of 2807 primary papers from 4 electronic databases. Of these, 2720 papers were excluded by the 2 investigators, leaving 87 papers remaining following the evaluation of the titles and abstracts (interrater agreement = 87%; kappa = 0.89). An additional 9 papers were identified after checking the references of relevant studies, resulting in 96 papers, which required full-text evaluation. After the full-text evaluation, 8 papers were included as a part of the final analyses.

### 3.2 Study characteristics

Table 1 provides the detailed study characteristics of the included studies. In the 8 included studies, 226 children with different degrees of airway obstruction were operated on in order to remove the tonsils or adenoids. In the included 6 studies, 135 nasal-breathing children with no airway obstruction were included in the control group, and the remaining 2 studies allocated 54 children with severe airway obstruction as control group.^[1,19]^ The number of children in each study ranged from 18 to 38. The age of the patients ranged from 3.4 to 15 years. The earliest study was published in 1974,^[11]^ whereas the most recent study was published in 2014.^[19]^ The follow-up period of the included studies ranged from 1 to 8 years. Three studies had a follow-up period of 1 to 1.5 years;^[1,11,19]^ another 3 studies had a follow-up period of 2 to 2.5 years;^[14,16,20]^ 1 study had a follow-up period of 5 years;^[21]^ and the remaining 1 had a follow-up period of 8 years.^[15]^ Of the 8 studies, 2 studies focused on the effects of adenoidectomy on children with airway obstruction,^[11,21]^ with another 3 studies focusing on the effects of tonsillectomy,^[1,14,16]^ and the remaining 3 studies focusing on the effects of both adenoidectomy and tonsillectomy.^[15,19,20]^ In terms of study design, all of the studies were prospectively clinical comparative studies. In terms of the geographic locations, 3 studies were conducted in South America^[16,19,20]^ and 5 in Europe.^[1,11,14,15,21]^

**Table 1:**
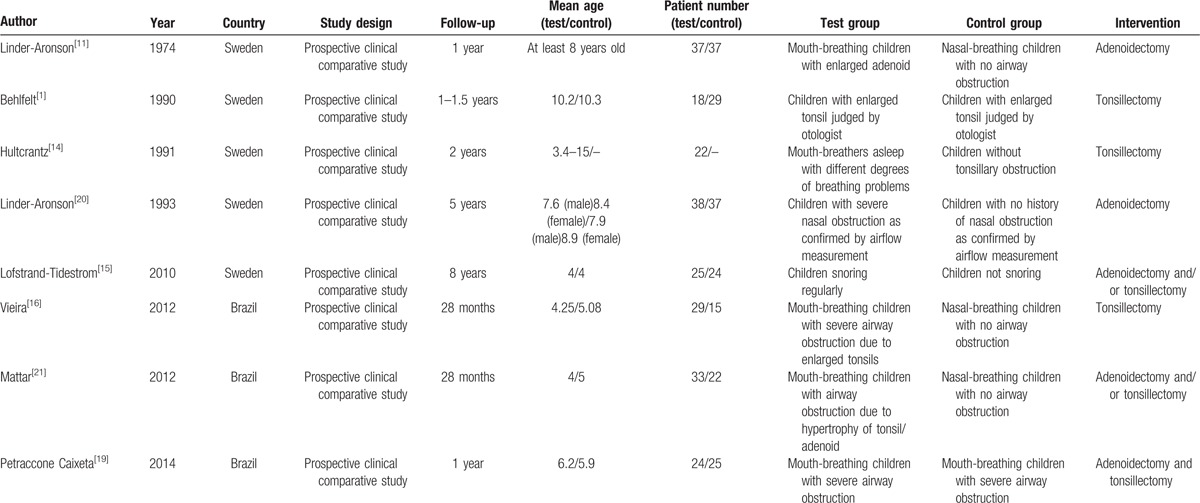
Characteristics of included studies.

### 3.3 Study outcomes

This systematic review and meta-analysis summarized the dental cast measurements from the 8 included studies. The outcomes of each included study are presented in Tables 2 and 3  .

**Table 2:**
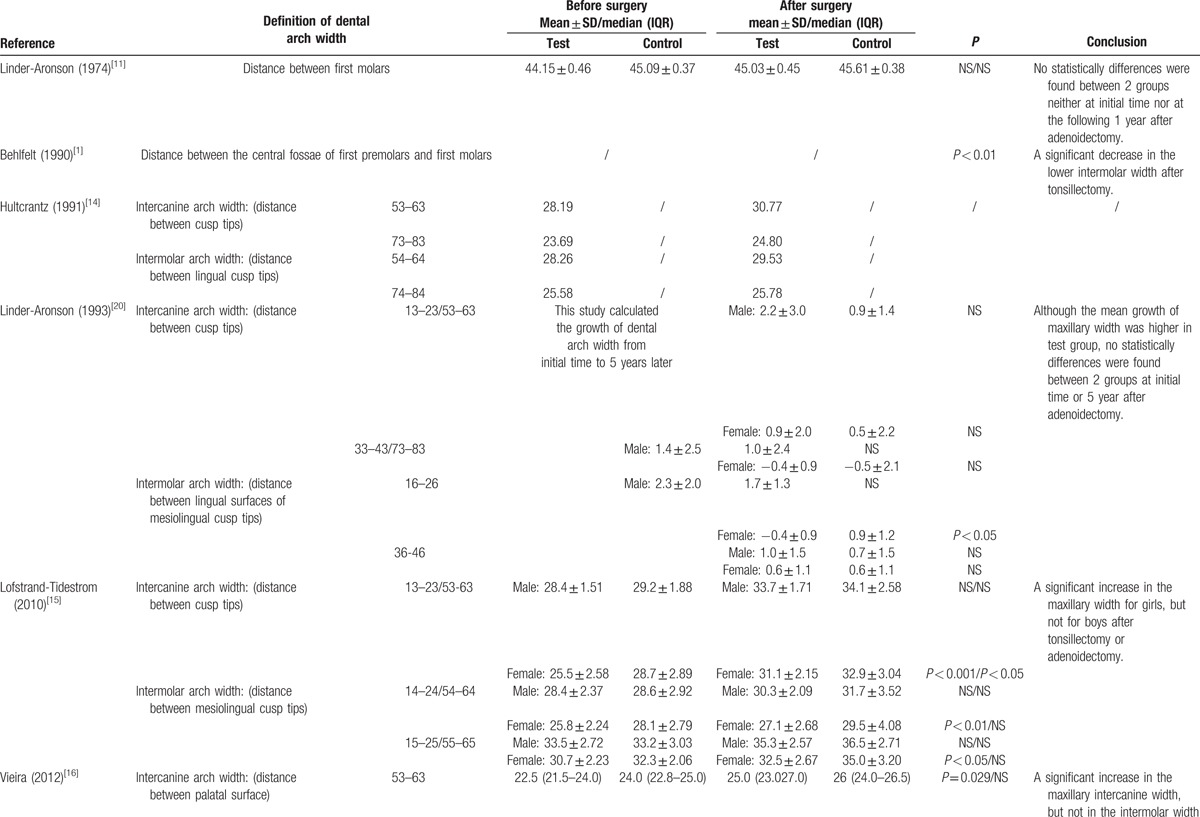
Detailed outcomes in dental arch width (mm).

**Table 2 (Continued):**
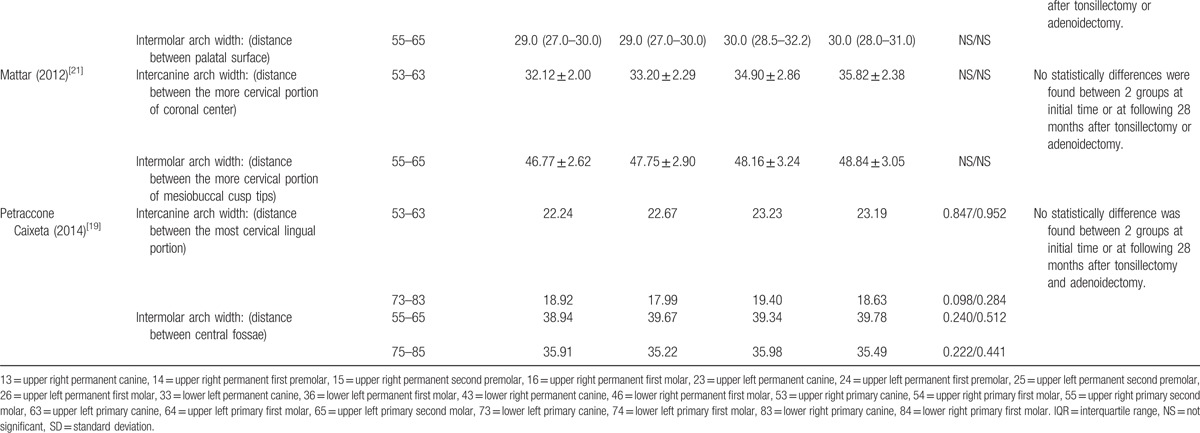
Detailed outcomes in dental arch width (mm).

**Table 3:**
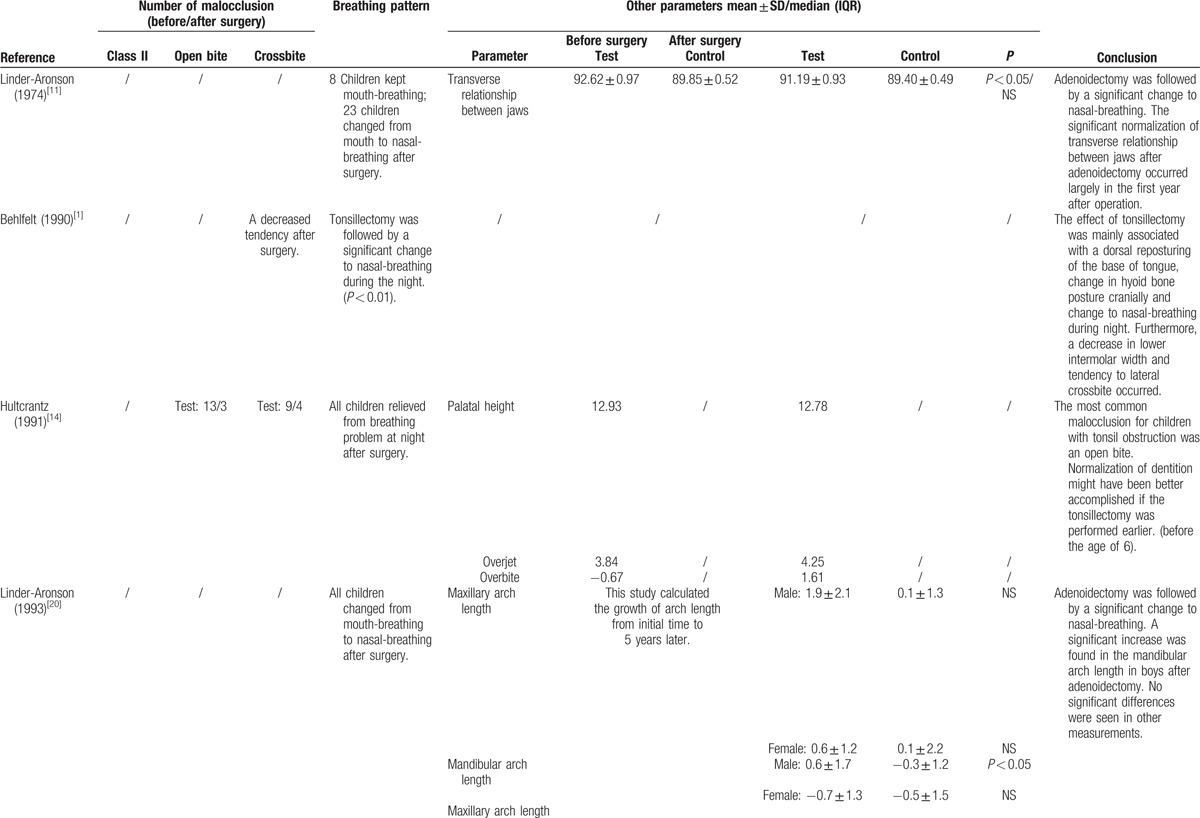
Outcomes of the included studies.

**Table 3 (Continued):**
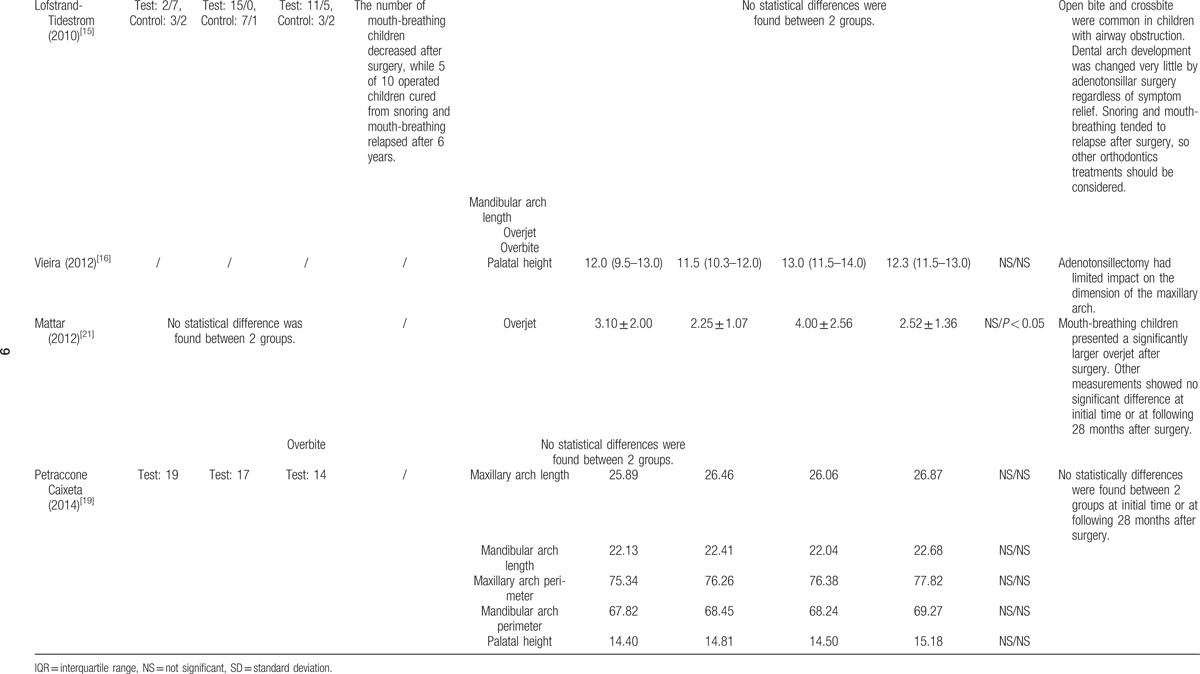
Outcomes of the included studies.

In terms of the limited number of studies, various definitions of test and control groups, as well as the diverse scope of topics discussed included as part of the selected studies, only a quantitative meta-analysis on upper dental arch width could be facilitated.

### 3.4 Dental arch width

The detailed measurements and data of each included study in dental arch width (mm) are presented in Table 2 . Eight studies were included. Three studies found no statistically significant difference between the surgical group and the normal group.^[11,20,21]^ One study found no statistically significant difference between the surgical group and the nonsurgical group with airway obstruction.^[19]^ In these studies, the increased dental arch width following the removal of airway obstructions was owed to child growth. Three studies observed a statistically significant normalization of dentition in the test group after surgery.^[1,15,16]^ Among them, 1 study observed a statistically significant increase in anterior upper arch width in children after tonsillectomy.^[16]^ One study observed a statistically significant increase in upper arch width only in girls, but not in boys, after adenoidectomy or tonsillectomy.^[15]^ One study observed a statistically significant decrease in lower arch width in children after tonsillectomy.^[1]^ Also 1 study observed an increase in dental arch width after a 2 year-follow-up, but no statistical analyses were employed to validate the observation's statistical significance.^[14]^

**Figure 2:**
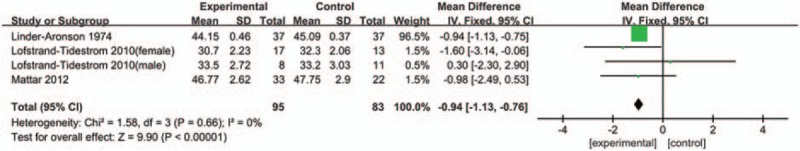
Forrest plots of the individual studies for the posterior maxillary dental arch width before surgery.

**Figure 3:**
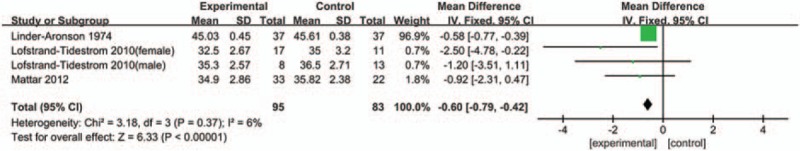
Forrest plots of the individual studies for the posterior maxillary dental arch width after surgery.

The results from meta-analyses are presented in Figs. 2 and 3. The results illustrated that children with airway obstruction had a significantly narrower posterior maxillary dental arch than nasal-breathing children without airway obstruction (WMD = −0.94, 95% CI [−1.13, −0.76]; *P* < 0.001). Low heterogeneity was evident among the included studies (*P* = 0.66, *I*^2^ = 0%). Following adenoidectomy or tonsillectomy, these children still had a significantly narrower posterior maxillary dental arch than nasal-breathing children (WMD = −0.60, 95% CI [−0.79, −0.42]; *P* < 0.001). There was low heterogeneity among the included studies (*P* = 0.37, *I*^2^ = 6%). Although the result from the meta-analysis after surgery was higher than the result before surgery. This illustrated a reduced severity of maxillary arch narrowing, with tendency to normalization evident in the maxillary dental arches in children with airway obstruction following the removal of enlarged tonsils or adenoids.

### 3.5 Change of breathing pattern

All of the 5 studies reporting the change of breathing pattern after surgery agreed that adenoidectomy and tonsillectomy were followed by a significant change from mouth-breathing to nasal-breathing.^[1,11,14,15,21]^ Two studies even reported that all of the participants were relieved of mouth-breathing and breathing problem at night,^[14,21]^ while 1 study found that relapse of snoring and mouth-breathing occurred following surgery during the follow-up period.^[15]^

### 3.6 Malocclusion

Open bite, crossbite, and angle class II malocclusion were reported to be the most common malocclusion in children with airway obstruction. Five included studies reported that children with airway obstruction had a higher risk of malocclusion.^[1,14,15,19,20]^ Among them, 3 studies found that a decreased tendency of malocclusion was evident after surgery.^[1,14,15]^ The total number of anterior open bite was reported to decrease from 28 to 10 in the surgery group of 2 studies.^[14,15]^ The total number of crossbite was reported to decrease from 20 to 7 in the surgery group of 2 studies.^[14,15]^ The number of angle class II malocclusion was reported to increase from 2 to 7 in the surgery group of 1 study.^[15]^ The number of crowding was reported to increase from 2 to 5 in the surgery group of 1 study.^[14]^ Meanwhile, 1 study reported that 2 groups presented statistically similar results concerning cross-bite, open bite, canine relationship, and primary 2nd molar terminal plane relationship neither at the initial time nor after surgery.^[20]^

### 3.7 Other parameters

#### 3.7.1 Palatal height

Palatal height (mm) was measured from the deepest point of the palate to a line connecting the mesial cusp of the deciduous 2nd molars^[16,19]^ or deciduous 1st molars.^[14]^ Three studies were included. No statistically significant difference was observed between the surgical group and the normal/nonsurgical group neither before surgery nor after surgery.^[14,16,19]^

#### 3.7.2 Overjet

The overjet (mm) referred to the distance between the incisal edge of the upper incisors and the labial surface of the lower incisors when the teeth were in centric occlusion.^[20]^ Three studies were included.^[14,15,20]^ One study reported, without the use of statistical analyses, that overjet tended to increase after 2 years following surgery.^[14]^ Another study found that the overjet in the test group increased significantly after 28 months following adenoidectomy or tonsillectomy.^[20]^ The other study found no statistical difference between the surgical group and the normal group in terms of overjet.^[15]^

#### 3.7.3 Overbite

The overbite (mm) referred to the distance between the upper incisal edge and the lower incisal edge when the teeth were in centric occlusion.^[20]^ Three studies were included.^[14,15,20]^ One study reported, without the use of statistical analyses, that overbite increased after 2 years following surgery.^[14]^ The other 2 studies found no statistical difference between the surgical group and the normal group in terms of overbite.^[15,20]^

#### 3.7.4 Maxillary and mandibular arch length

The dental arch length (mm) was measured from the midpoint between central incisors to the tangent line to the distal surface of the right and left deciduous 2nd molars^[19]^ or mesial surface of the permanent molars.^[21]^ Three studies included this measurement.^[15,19,21]^ All 3 studies reported a trend toward increased maxillary arch length after surgery in the test group, while no statistically significant difference was observed between the surgical group and the normal/nonsurgical group neither before surgery nor after surgery. Similarly, in terms of mandibular arch length, no statistically significant difference was observed between the surgical group and the normal group, excepting for the statistically significant increase evident in male participants following adenoidectomy in 1 study.^[21]^

#### 3.7.5 Maxillary and mandibular arch perimeters

The perimeter of the dental arch (mm) was measured from the distal surface of left deciduous 2nd molar to the distal surface of right deciduous 2nd molar passing over the central fossae of deciduous molars, the tip of the deciduous canine, and the incisal edge of the incisors. This value denoted the shape of the dental arch. One study included this measurement. No statistically significant difference was found between the surgical group and the nonsurgical group with airway obstruction, neither at the initial time nor at the following 28 months after surgery.^[19]^

#### 3.7.6 Transverse relationship between jaws

The ratio of transverse relationship between jaws = (the width of lower jaw between 1st molar/the width of upper jaw between 1st molar) × 100%. Index values close to 100% indicated cusp-to-cusp dentition. A high index value indicated a tendency to cross-bite. One study calculated this index. The result showed a decreased, and normalized tendency of the transverse relationship between jaws after surgery. This change proved to be statistically significant.^[11]^

### 3.8 Publication bias

Publication bias was determined by the visualization of funnel plots. The funnel plots are presented in Online Resource (Figures 1 and 2, Supplemental Digital Content).

### 3.9 Quality assessments

The result of quality assessments is presented in Online Resource (Table, Supplemental Digital Content). Of the 8 included studies, 2 studies were estimated to have a moderate risk of bias.^[14,20]^ The remaining 6 studies were estimated to have a low risk of bias.^[1,11,15,16,19,21]^

## 4 Discussion

A recent meta-analysis has proved that children with severe airway obstruction have a retrognathic mandible, a vertical direction of growth, and a tendency toward class II malocclusion.^[22]^ For children with severe airway obstruction or obstructive sleep apnea syndrome, adenoidectomy and tonsillectomy were recommended. It was hypothesized that the removal of obstructions had a role in relieving the oropharyngeal airway passage. This would allow for the normalization of tongue and hyoid bone postures, and would thus facilitate nasal breathing. Balance of function and soft tissue might inhibit or even reverse hard tissue growth.^[1,23,24]^ In recent times, a trend toward a more restrictive criteria for adenoidectomy and tonsillectomy was evident; owing to a paradigmatic shift toward the view that the tonsil and adenoid, as lymphatic organs, played an important role in the development of primary immunologic defense system.^[25,26]^ Additionally, it was increasingly perceived that the procedures did not warrant the risk of complications associated with surgical and postsurgical conditions.^[27–29]^ Therefore, orthodontists and otolaryngologists need more convincing evidence to prove that adenoidectomy and tonsillectomy will have a positive effect on children with airway obstruction, implicated in incidences of mouth-breathing and dental deformity.

Nonetheless, the debate concerning the role played by the adenoidectomy or tonsillectomy-mediated reversal of dental deformities in cases of airway obstruction in children is still ongoing. Therefore, the aim of this meta-analysis and systematic review was to study the effect of adenoidectomy and tonsillectomy on dental arch morphology in children with airway obstruction.

This systematic review utilized 8 different studies. Four of which, verified that adenoidectomy or tonsillectomy could contribute to the normalization of dentition in children with enlarged tonsils or adenoids that obstructed their airway.^[1,11,14,19]^ The remaining 4 studies indicated that the surgery had a limited impact on dental arch dimensions, and furthermore, supported that genetics probably had the greatest impact on dentition.^[15,16,20,21]^ The result from the meta-analysis of posterior maxillary width illustrated the normalized tendency of dental arch width following the removal of obstruction, but no statistical significance was found between the surgical and the normal group.

In terms of sexual dimorphism, 2 studies illustrated that adenoidectomy or tonsillectomy had a more beneficial effect for females than males in terms of airways passage enlargements.^[15,21]^ Linder-Aronson study found that, in females, a greater proportion of pharyngeal space was occupied by lymphoid tissue.^[30]^ Thus, adenoidectomy and tonsillectomy would have deeper implications for girls. In terms of the recommended age for surgical intervention, 2 studies indicated that normalization of dentition might have been better achieved if the surgery was conducted before the age of 6, as the compensatory changes in dentition growth did not appeared to be permanent.^[16,21]^ After children developed mixed dentition, it was more unlikely to get a spontaneous dentition correction following surgery.^[31]^ In terms of relapse, 1 study reported that snoring and mouth-breathing tended to relapse after surgery.^[15]^ The relapse was due to the habit of open-mouth posture, regrowth of adenoid tissue, and primary craniofacial deformity.^[32,33]^

Through a wide search of the relevant studies and summarizing the included literature, current research into this field proved to be insufficient and had several defects. First, in the 8 included studies, only 2 of the study designs considered children with severe airway obstruction within the context of nonsurgical patients as the control groups.^[1,19]^ All other studies compared children with airway obstruction who underwent surgery relative to nasal-breathing children with no airway obstruction due to clinical limitations. This weakened the correlative strength of the evidence. Moreover, the patients in the control groups should be matched by not only chronological age, but also by the stage of skeletal maturation via lateral cephalometric radiography, against those patients in the test groups.^[19]^ Most of the included studies only matched the control groups for patient age and sex against that of the test groups. Second, the measurements of dental arch dimension should be improved upon. As indicated by Petraccone study, the results of dental arch width lacked statistically significant difference between the surgical group and the nonsurgical group with airway obstruction. This was evident both prior and after surgical intervention. However, when dental arch dimensional changes were converted into percentile changes because of the head size of children varying even in the same developmental stage, the maxillary arch width of the test group showed significant increases relative to that of the control group.^[19]^ This conversion illustrated that the percentage change between the initial recording and the recording done several years postsurgery could be considered as a better measurement for estimating differential growth potentials between test and control groups, respectively. A measurement getting rid of other confounding factors is needed in future studies. Third, the definition of dental arch width was not standardized across studies. Thereby, it was difficult to merge the results from different studies and would introduce the heterogeneity. In conclusion, more high-quality and well-designed clinical trials with larger sample sizes, comparable control groups, standardized definitions, and more precise measurements are needed in future studies.

Moreover, before concluding that adenoidectomy or tonsillectomy had limited effects on the normalization of dental morphology once dental deformity had formed, the following factors must be considered: whether the follow-up time was long enough to evaluate the dental arch dimension changes after surgery, and whether the time of surgery was too late for children with narrow dental arches caused by mouth-breathing to get reversible changes of dental morphology. Owing to the limited number of included studies, a subgroup analysis could not be conducted to illustrate the influence of follow-up period and surgery time relative to the results. These 2 questions are still under ongoing debate.

To our knowledge, this systematic review is the most comprehensive study examining the effects of adenoidectomy and tonsillectomy on dental arch growth in children with airway obstruction; however, there were still some limitations. First, it was difficult to completely eliminate the confounding factors inherent in the included studies, which may have resulted in a bias toward the outcomes. Second, as a consequence of the clinical and ethical limitations, randomized controlled trials were not available in the provision of high-quality evidence. Third, the literature search was limited to studies in English, and only covered 4 electronic databases, which may have resulted in an election bias toward this study's outcomes. Therefore, in considering the aforementioned limitations, the results of this meta-analysis should be interpreted with caution.

## 5 Conclusion

The included studies failed to establish definitive statistical power for the normalization of dental arch dimensions following adenoidectomy or tonsillectomy in children with airway obstructions. As the effect of surgery was limited, other treatments such as functional training or orthodontic maxillary widening should be considered following removal of obstructions in the airway.

## Acknowledgments

We would like to thank Editage (http://www.editage.cn) for English language editing.

## Supplementary Material

Supplemental Digital Content
