# Supplementary material for: Dental arch dimensional changes after adenoidectomy or tonsillectomy in children with airway obstruction: A meta-analysis and systematic review under PRISMA guidelines
Source: Medicine (Baltimore). 2016 Sep 30;95(39):e4976. doi: 10.1097/MD.0000000000004976 (PMC5265940; doi:10.1097/MD.0000000000004976)
Supplement: Supplemental Digital Content [file medi-95-e4976-s001.docx]

Supplemental Digital Content.

Figure 1 That demonstrates the funnel plot of the posterior maxillary dental arch width before surgery.


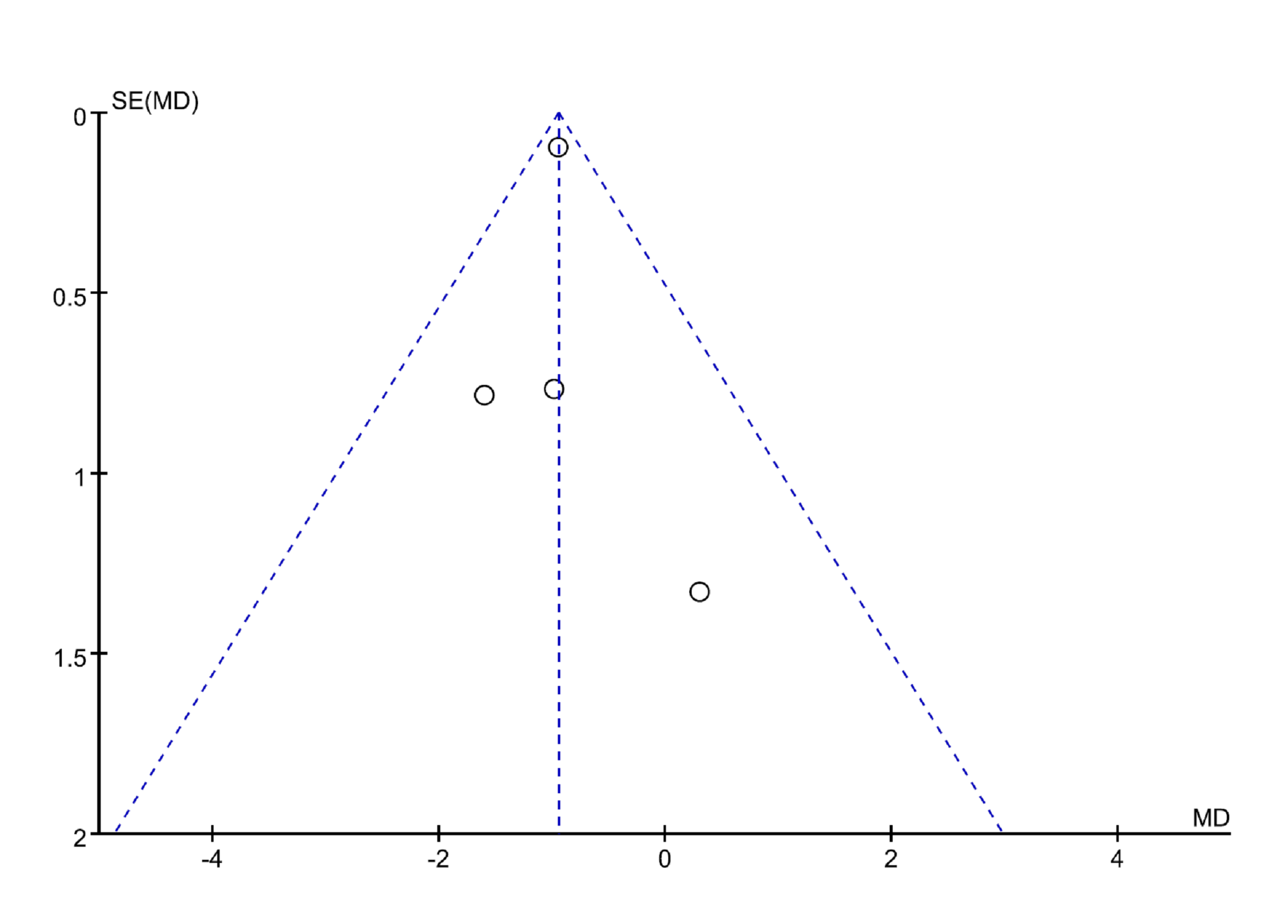


Figure 2 That demonstrates the funnel plot of the posterior maxillary dental arch width after surgery.


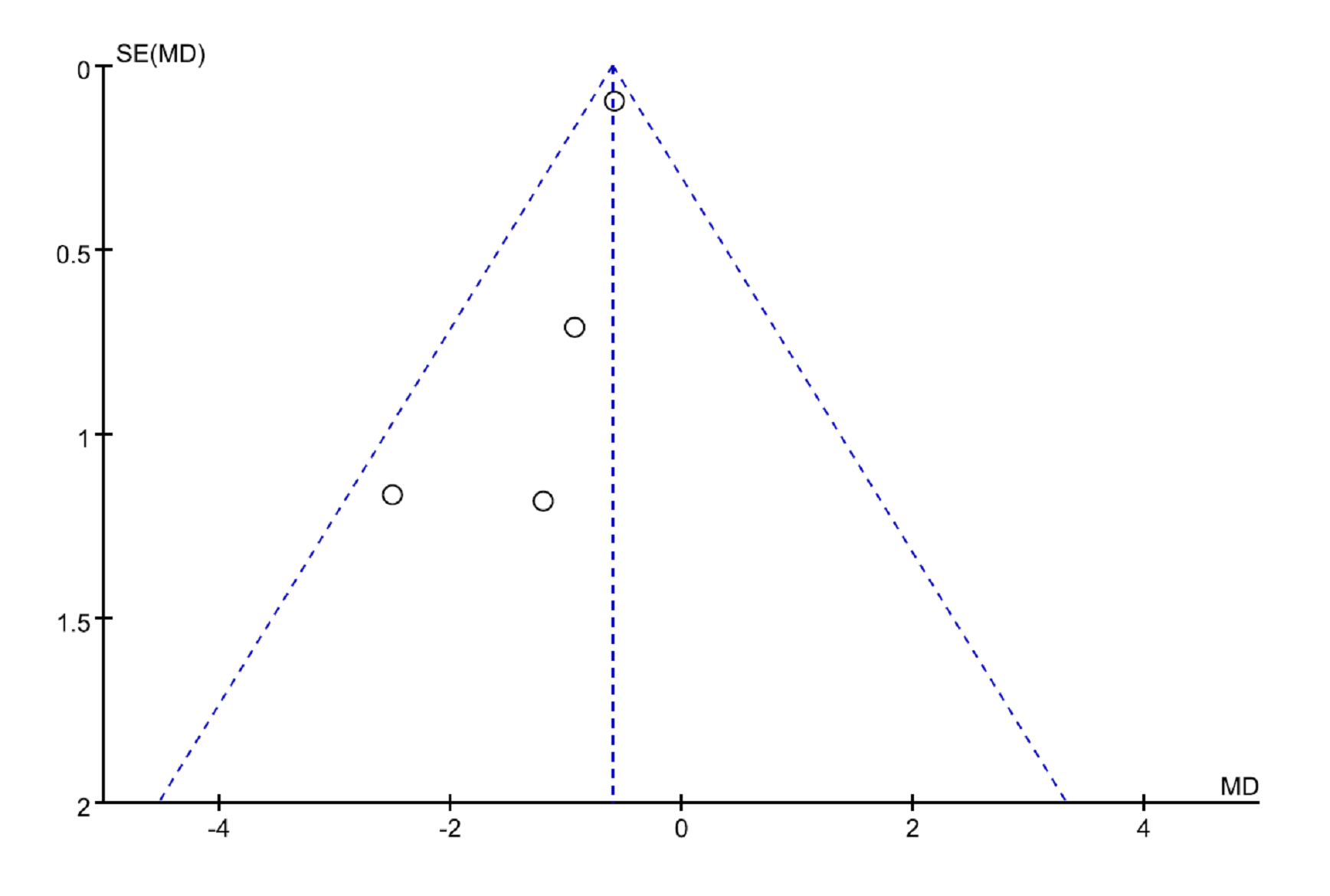


| **Table. Quality assessment of included studies** | | | | | | | | | |
| --- | --- | --- | --- | --- | --- | --- | --- | --- | --- |
| **Author** | **Selection** | | | | **Comparability** | **Outcome** | | | **Total score** |
|  | Representativeness of the exposed cohort | Selection of the non-exposed cohort | Ascertainment of exposure | Demonstration that outcome of interest was not present at start of study | Comparability of cohorts on the basis of the design or analysis | Assessment of outcome | Was follow-up long enough for outcomes to occur | Adequacy of follow up of cohorts |  |
| Linder-Aronson  (1974) | ***** | ***** | ***** |  | ND | ***** | ***** | ***** | 6 |
| Behlfelt(1990) | ***** | ND | ***** |  | ***** | ***** | ***** | ***** | 6 |
| Hultcrantz  (1991) | ***** |  | ***** |  |  | ***** | ***** |  | 4 |
| Linder-Aronson  (1993) | ***** | ND | ***** |  | ****** | ***** | ***** |  | 6 |
| Lofstrand-Tidestrom  (2010) | ***** | ***** | ***** |  | ND | ***** | ***** | ***** | 6 |
| Vieira  (2012) | ***** |  | ***** |  | ***** | ***** | ***** | ***** | 6 |
| Mattar  (2012) | ***** | ***** | ***** |  | ND | ***** | ***** |  | 5 |
| Petraccone Caixeta  (2014) | ***** | ***** | ***** |  | ****** | ***** | ***** | ***** | 8 |
| ND = no description. | | | | | | | | | |
